# Extensive pemphigus vegetans in a Filipino female

**DOI:** 10.1016/j.ijwd.2021.06.004

**Published:** 2021-06-21

**Authors:** Maicka Keirsten O. Agon, Danielle Nicolle Dionisio Mejia, Catherine Anne Pacis Cifra, Mae Ramirez-Quizon

**Affiliations:** a Department of Dermatology, Rizal Medical Center, Manila, Philippines; b Department of Dermatology, Philippine General Hospital, Manila, Philippines

**Keywords:** Pemphigus vegetans, rare, verrucous, vegetating, extensive

**Fig. 1:**
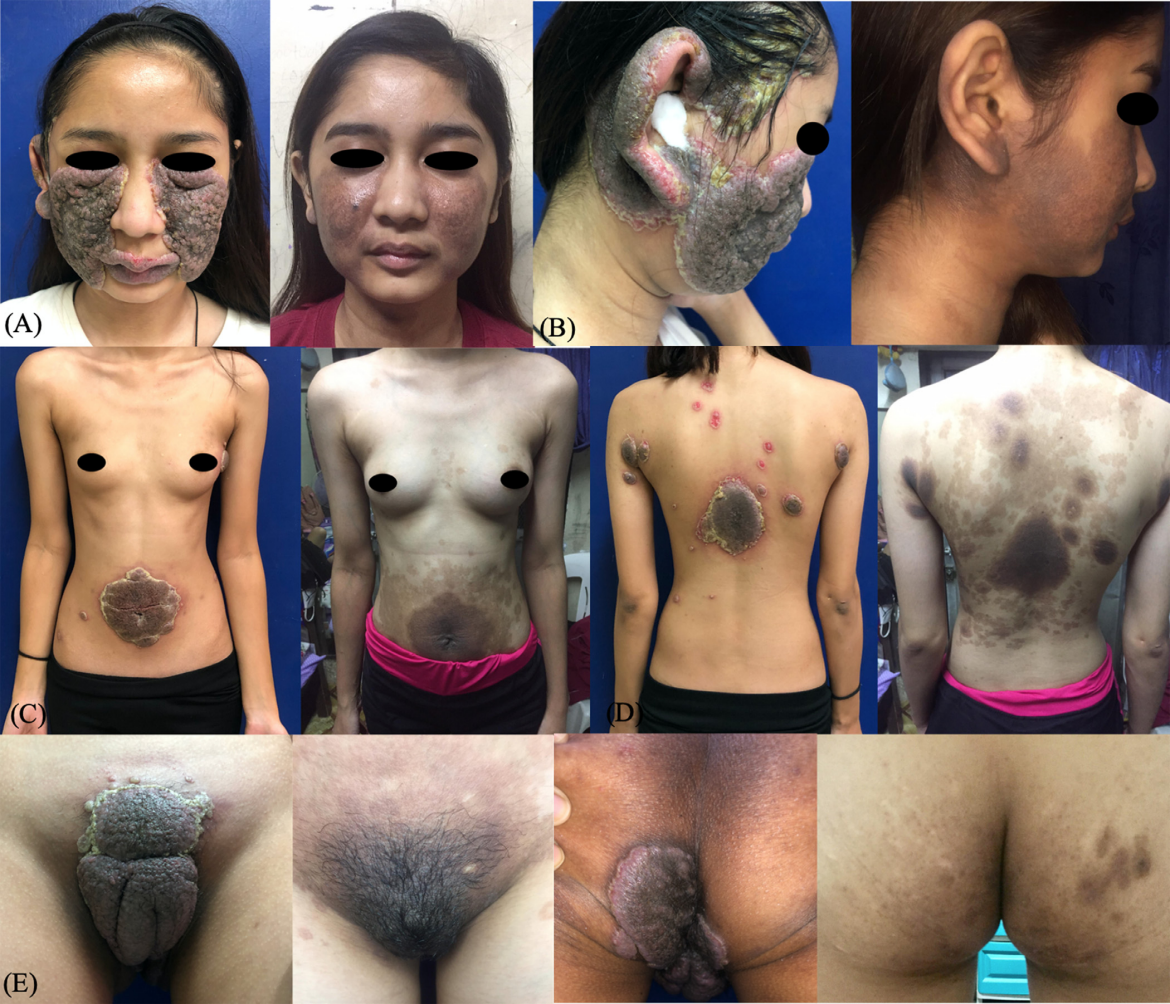
Initial and treatment response after 19 months. Initial cutaneous examination revealed multiple round hyperpigmented vegetating plaques with pustules and purulent discharge and crusts around its borders at the bilateral cheeks extending to the lower border of the (A) eyes and lips; (B) right ear and parietal scalp; (C) trunk; (D) back; and (E) anogenital areas. After 19 months of prednisone and azathioprine, the patient had a follow-up consultation via teledermatology, leaving multiple, well-defined, hyperpigmented patches over the same sites.

**Fig. 2:**
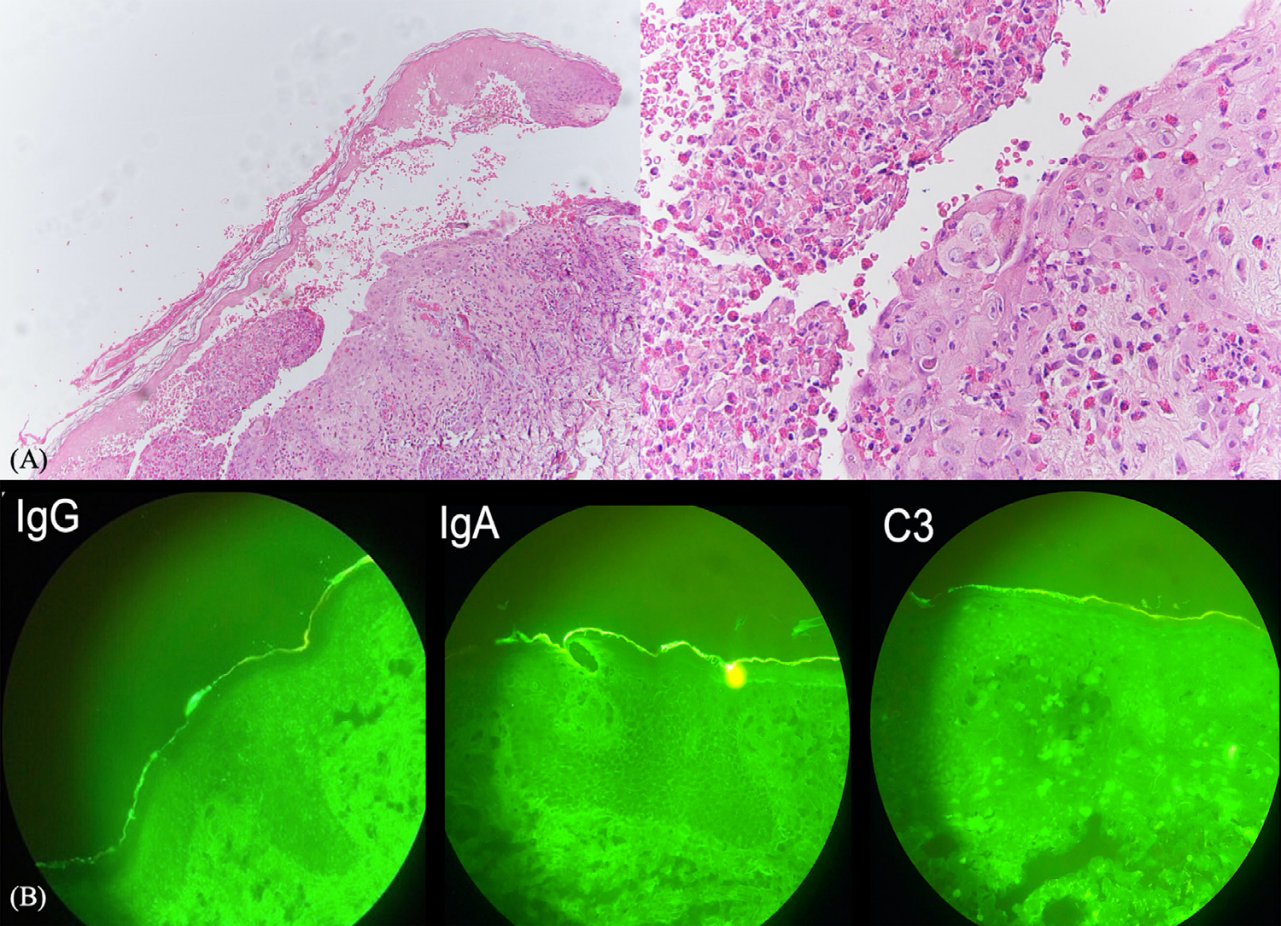
(A) Histopathology showed an intraepidermal split, acantholysis, pseudoepitheliomatous hyperplasia, mild superficial perivascular dermatitis, and markedly dense infiltrates composed of lymphocytes, eosinophils, and histiocytes. (B) Direct immunofluorescence revealed faint intracellular deposits of IgG, C3, and IgA. All are consistent with Pveg.

Dear Editors,

Pemphigus vegetans (Pveg), a clinical variant of pemphigus vulgaris accounting for 1% to 2% of all cases, is a rare autoimmune blistering disease characterized by flaccid pustules, vesicles, or bullae that erode and form hypertrophic vegetative plaques with vesicles or pustules at the periphery of these lesions (Matsuyama et al., 2018). We report a case of an 18-year old Filipino woman who initially presented with a 5-month history of oral ulcers and vegetating hyperpigmented pruritic plaques, which increased to extensively involve the face and body a month later (Fig. 1). Differential diagnoses at that time were verrucous carcinoma, tuberculosis verrucosa cutis, and pyoderma vegetans. Histopathologic findings revealed an intraepidermal cleft with acantholysis, pseudoepitheliomatous hyperplasia, mild superficial perivascular dermatitis, and markedly dense infiltrates composed of lymphocytes, eosinophils, and histiocytes (Fig. 2). Direct immunofluorescence showed intercellular deposits of IgG, C3, and IgA (Fig. 2). Enzyme-linked immunosorbent assay revealed elevated levels of desmoglein 3 (36.63 RU/mL) and desmoglein 1 (9.80 RU/mL). The patient was started on oral prednisone at 40 mg/day and azathioprine 100 mg/day, which showed marked improvement after 4 weeks of treatment.

In the literature, Pveg lesions are mostly seen localized on flexural or intertriginous areas and oral mucosa (Matsuyama et al., 2018). Few case reports involved unusual areas, such as the nails, arm, trunk, scalp, and esophageal sites (Messersmith and Krauland, 2021). In some areas, features of Pveg may be specific, such as the cerebriform tongue (sulci and gyri pattern on the dorsum of the tongue), cobblestoning of the lips, and verrucous paronychia of the nails (Suwarsa et al., 2017). Our case involved extensive involvement of Pveg of the face, ears, scalp, trunk, back, genital, and gluteal area. A similar report of a 30-year old female patient with extensive involvement of the mucosa, trunk, extremities, verrucous paronychia, and fissure tongue was published in 2019 (Suwarsa et al., 2017), albeit with less extensive involvement compared with our patient.

To date, the mechanism of the formation of verrucous vegetations from blistering lesions remains unknown and its exact pathogenesis is still unclear (Messersmith and Krauland, 2021). It may result from relative occlusion and maceration with subsequent bacterial infection, which may explain the vegetations in intertriginous areas but cannot explain the lesions in non-intertriginous areas (Verma et al., 2019; Zaraa et al., 2011). Another mechanism could be immunopathologic factors, such as the Th2-mediated immune reaction (interleukin-4) with involvement of IgG4 to IgG2 autoantibody isotypes and cytokines, which play a role in epithelial proliferation and eosinophil chemotaxis (Zaraa et al., 2011) and elevation of eosinophilic cationic factor and TGF-alpha that could account for the epithelial cell migration and proliferation, adding to the appearance of vegetating lesions (Verma et al., 2019; Zaraa et al., 2011). Isolated case reports of factors contributing to Pveg include HIV infection, intranasal heroin abuse, enalapril, captopril, and organ transplant (liver, kidney; Verma et al., 2019; Zaraa et al., 2011).

Due to its rarity and variable presentation, Pveg may be difficult to diagnose initially; hence, a high index of suspicion is vital when vegetating lesions with mucosal lesions are present. To our knowledge, our case exhibits the most extensive lesions of Pveg in the literature that was successfully treated with oral corticosteroids and immunosuppressant.
